# Is the Germ Theory of Disease Rational?

**Published:** 1888-05

**Authors:** J. S. Todd

**Affiliations:** Atlanta, Ga., Professor of Materia Medica and Therapeutics in the Atlanta Medical College


VOL. V. MAY, 1888. NO. 3.

### Original Communications.

### IS THE GERM THEORY OF DISEASE RATIONAL?*

BY J. S. TODD, M. D., ATLANTA, GA., PROFESSOR OF MATERIA MEDICA AND THERAPEUTICS IN THE ATLANTA MEDICAL COLLEGE.

Dr. A. L. Loomis, in his address before the New York Medical Society, says: "The only disease, in which a bacterial origin has been completely demonstrated *in man* (italics mine) are erysipelas, splenic and relapsing fever." "The proof," says he also, "that the tubercle bacillus is the cause of tuberculosis requires yet the crucial test of human inoculation." It is a well-known fact that some of the lower animals are not affected in the same manner by medicines as man, and also that they enjoy immunity from certain diseases that affect the latter; for instance, the monkey cannot be given syphilis by inoculation. This, it seems to me, is a fair test, if it be demonstrated that there was a reproduction—multiplication of the inoculated germs taken from man and injected

*Read before the Medical Association of Georgia, April, 1888.

into the inferior animal. At the same time the animal suffering with the disease which the human being who furnished the germ had. There is no need for the crucial test in tuberculosis, for almost all the lower animals are subject to tuberculosis. But the admission by so distinguished a physician as Dr. Loomis, that it has been demonstrated that germs are the cause of any disease, is a hopeful and encouraging sign. Dr. Loomis is nearly 60; men over 40 rarely change their opinions, or even take the trouble to investigate new theories. The late Dr. Flint, however, was an enthusiastic advocate of bacteriology; he, by almost universal consent, was regarded as the first of American physicians. Dr. DaCosta, a little Dr. Loomis' junior in years, says in his "Diagnosis" (last edition), a book that has made his name famous around the globe, and that is read in many tongues, after giving a cautious but general assent to the bacillus tuberculi being present in tuberculous sputum, "not finding them, is not as conclusive evidence as finding them, for Koch failed to detect them in a certain number of cases of consumption." The acceptance by Dr. W. F. Westmoreland, Sr., of antisepsis in the practice of surgery gives another proof of his right to the proud title of Georgia's greatest surgeon. Always a searcher after truth, seeing the truth, he was not ashamed to put it in actual practice, if it did conflict with his earlier views. Thank God for these rare old dogs that can be taught new tricks. I endorse the following from Dr. Weir, of New York: "Nor is cleanliness the whole matter, for, if you trust to that alone, the merest tyro in surgery, who uses antiseptics, will far outstrip you in his recoveries." It is claimed that specific microbes have been found in the diseases mentioned below: Erysepilas, tuberculosis, small-pox, syphilis, measles, diphtheria, scarlet, splenic, malarial, relapsing, typhus, typhoid and yellow fevers, cholera, pneumonia, pyemia, leprosy, acute rheumatism, ulcerative endocarditis, pyelitis, gonorrhœa, atrophy of the liver, and numerous skin diseases, such as sychosis, etc. Now it has not been demonstrated that such is the fact—that is, that specific germs are the cause of each and every one of the diseases above mentioned, but it is only claimed that such is the case, and I distinctly disclaim my disbelief of their presence in all the diseases named. I do believe, however, that all contagious or infectious maladies are caused by germs. I am no microscopist, but make the above assertion because of the following reasons: Take syphilis or small-pox for example: the minutest quantity conceivable inoculated of either of the viruses will reproduce their kind. A portion imponderable, so to speak, floating in the atmosphere and wafted for considerable distance, of small-pox virus causes variola in those susceptible. Now they are not poisons, for poisons are inorganic substances that enter into new combinations with the tissues, and act toxically only when given in excessive doses; they are practically destroyed in destroying; they do not reproduce their kind; for instance, if a man was killed by a dose of two grains of arsenic, another man, to be poisoned by the same arsenic, would not only have to turn cannibal, but would have to eat all parts of the man so poisoned at one meal, and even then the new combinations that arsenic would form with the iron, albumen, etc., in the poisoned individual's blood and tissues would, in all probability, have rendered the arsenic inert ; yet, from an almost inconceivably small dose of syphilitic or vaccine virus you get a quantity sufficient to reproduce them in thousands. Again: a man once poisoned with a drug it does not give him immunity from its toxic effect in future; as a rule, on the contrary, those who have suffered from contagious diseases enjoy immunity afterwards ; the pabulum upon which the germs feed has been exhausted. Variolus or syphilitic virus kept for a length of time, be the precautions taken what they may to preserve them, lose their power of infecting. Arsenic and other mineral poisons, gotten from the deepest strata of the oldest rocks and preserved from the remotest time, do not lose their power to destroy. The one was a vital something that, obeying the laws of nature, lived out its allotted time and died; the other, never having had life, could not die. Vaccine virus—a crust of it—immersed for a short time in a solution of corrosive sublimate, one part to the 1,000, loses its power of communicability ; the living something in it is killed by even the minute quantity of this great germicide. In the treatment of small-pox, pitting is best avoided

(and this has been known for ages) by the local application of mercurial ointment. Now what is the philosophy of this action, what its *modus operandi ?* The fumes of mercury destroy the life in seeds; they do not germinate; the ovum of the egg even is killed by it. (Stille says: "It checks the development of the small-pox pustules and thereby tends to prevent the pitting.") How? Not by any local antiphlogistic, astringent or sedative effect surely, for it causes irritation when long applied. No other rational explanation can be offered, it appears to me, than this: Each pustule is a local foci for a number of germs; mercury kills them before they fully develop; it eliminates the cause which leads to the local destruction of skin tissue. Its use is of course limited to the face, for applied to the entire cuticle there would be danger of salivation, and its application to the mucous membranes that are affected in this disease would be impracticable or, if possible, fatal. The highly inflamed condition of the skin so impairs its power of absorption that salivation is not feared, and with a limited application to the face no ill result has occurred.

Mercury is the best-known remedy in syphilis—is almost a specific. No explanation of its antidotal power is so simple as its germidal. The assertion that a sufficient quantity of corrosive sublimate to kill the syphilitic corpuscle or germ would prove fatal to the individual is capable of qualification. The germ has not yet been demonstrated—isolated (although I believe it does exist). Still we do not know that such is the case—that it would thrive or live in so minute a quantity of corrosive sublimate as is necessary for the cure of the disease in question. But granting, for the sake of argument, the truth of the assertion, that by no means leads inevitably to the conclusion that the antidotal theory is to be abandoned. Fish live for a length of time in polluted streams; they will feebly reproduce for a short while, but inevitably, the pollution continuing, they die. The effect of mountain air in giving back the blush to beauty's cheek is not immediate; the unwholesome atmosphere of a dungeon does not prostrate at once; men live for months surrounded by marsh miasma in perfect health. Heat and moisture are necessary to develop malarial diseases. Numbers of physicians claim to have discovered the specific cause of malaria. I am not prepared to say it has been demonstrated, but that it exists I believe, and that quinine is the antidote I am equally sure.

Several independent, thoroughly-trained workers in different quarters of the globe, using in their researches the lenses of the best opticians, have arrived at the following conclusions: In the blood of malarial subjects an organism is invariably found which is absent in all other conditions. 2d. With the improvement in the condition of the patient the parasite disappears or remains in but one of its protoplasmic forms—the pigmented crest. 3d. Quinine, the universal specific for malarial poisons, is fatal to this parasite, so much so that it is a *sine qua non* that in the search for the organism the patient shall not have recently taken the drug. Professor Councilman, of the John Hopkins University of Baltimore, confirms the above conclusions in a recent lecture before the Pathological Society of Philadelphia. Professor Osler, of the University of Pennsylvania, in a spirit of scientific incredulity, began experiments to disprove Councilman's conclusions which resulted in his conversion to the views of the latter.

We are often asked the question by the older physicians, " How about some diseased germs reproducing, with such marvelous rapidity, inconceivable multiplication (say they with a sneer), while others, as tubercle bacilli, for instance, have been twenty-five, thirty and fifty years in developing? In answer to the above question, the following quotation from Brunton's Therapeutics is so appropriate and so fully and completely expresses my views that I beg no pardon for its length:

"PROBABLE MODE OF ACTION OF ARSENIC IN PHTHISIS."

"The treatment of phthisis is so important that it may be advisable to discuss in a few words the probable mode of action of arsenic and hypophosphites in its early stages. It is now probable that this disease depends on the presence of a bacillus (B. tuberculosis, p. 90).

"In order that it should grow within the body, however, it is necessary that a suitable nidus should be present, and the different susceptibility to the disease of different individuals, or of the same individual at different times, probably depends on their liability to present a suitable nidus. The bacillus tuberculosis differs from such bacilli as the B. anthracis in being of very slow growth, so that when it is cultivated artificially on a solid medium it takes about ten days before it succeeds in establishing itself and begins to grow. Consequently, when applied to an open wound, or when inhaled into the lungs of a healthy person, it does not, like the bacillus anthracis, at once begin to multiply and produce disease in the organism, but it is usually removed by washing, in the case of a wound, or by expectoration in healthy persons. But if its removal be interfered with, it will produce the disease. Thus if, instead of being applied to an open wound, it be injected under the skin, so that it cannot be removed by washing, it will, after a time, begin to grow, and produce tuberculosis, first local and then general. It is probable that the case is similar in the lungs. In the healthy lung it finds no nidus, and is removed by expectoration; but if a portion of the lung be consolidated by catarrhal pneumonia, the consolidated part probably affords a nidus to the bacillus, and the longer the consolidation lasts the greater the risk of bacilli finding entrance. In croupous pneumonia the exudation into the alveoli, consisting chiefly of fibrin with a few leucocytes, quickly breaks up and is absorbed, so that it is comparatively rarely followed by phthisis. But the proliferated epithelial cells which fill the alveoli of the lung in catarrhal pneumonia are much more resistant, they break down and are absorbed much more slowly, and hence a much longer time is given during which bacilli may find a nidus. The marked hereditary nature of phthisis is a curious point in a disease which we suppose to depend on the presence of a bacillus, and is a character in which it differs from such diseases as anthrax, ague, or relapsing fever, which are also due to foreign organisms. But the difference probably depends on the slow growth of the tubercle bacillus, which renders a prolonged undisturbed rest at the point where it enters the body necessary for its farther growth.

*The disease is not hereditary, but the predisposition* to such morbid changes in the lungs as afford a nidus to the bacilli *is hereditary.*" My preceptor, Dr. A. W. Griggs, taught me twenty years ago that consumption was not hereditary.

Again, we are often asked, "How is it that if water contains germs which occasion, and only occasion, typhoid fever, is it that in mountain districts we often find it in localities widely separated, where there are no sewers, sewer-gas or sewerage that can make pollution?" The question takes many things for granted. We simply answer, explain this fact. Simultaneously, in almost all the middle portion of Georgia, there sprung up, as if by magic, over the face of the earth, pigeon-clover? We scientific men will not say it was a spontaneous generation. Each bunch had a progenitor exactly like it. The negroes say Sherman brought it with him. Well, if he did, he did not intend to, I am sure. Ruin, fire, famine and desolation marked his vandal path! A sower of salt, not seed, was he. If clover-seed, which is not microscopic, is thus wafted by the winds, why not germs, and what hinders their falling into wells, branches, springs, etc.?

"Marquelle," says Dr. Weir, "has found that the ordinary atmosphere of a large city contains over two thousand bacteria per cubic yard, while the air of a room in an old house in winter will show bacteria to the amount of forty-five thousand per cubic yard; and again, that the walls of a long-used hospital will hold as many as ninety thousand in the same space."

With the crucial test of clinical demonstration staring any one, who will look, in the face, no man having any regard for his veracity can doubt the practical good results that have accrued to surgery from the use of antiseptics. Dissertations to prove that antisepsis is only cleanliness; that it is useless or unnecessary at this late day, reminds me of the learned (?) English philosopher who wrote a volume to prove that steam could never be applied to locomotion. That same book was brought to this country in a vessel propelled by steam.

The reduction in the number of infectious diseases that so closely and invariably follow the improvements in the water supply of our large cities is an argument and a practical demonstration of the fact that something was excluded which was deleterious.

To say that epidemic and so many other diseases are caused by cold, telluric or atmospheric influences—words which cloak our ignorance and savor of superstition—are terms which are being restricted before the positive, demonstrable, living presence of germs as etiological factors. The immunity from cholera, which was enjoyed by all save one of the German physicians while visiting India to investigate this disease, carries with it a lesson. They searched for it where it was most prevalent, literally lived in its atmosphere, walked among its victims, handled them, touched it, felt them, investigated it with an intimacy which made contagion the most possible; yet the only precaution they took was the drinking of boiled water. But one of their number had the disease, and he attributed it to drinking water that was not boiled. It appears to me that the heat destroyed a living something—bacillus, microbe or whatever you may choose to call it—that is the cause of the disease.

Now let us see about the best recognized treatment for an umber of diseases, Fresh air, freedom from germs, or germs largely diluted, cleanliness in person and surroundings—in other words, depriving bacteria of a nidus—pure water (impure being the source of many, very many diseases.)

Now for drugs. Nine-tenths of all the useful and generally used ones are germicidal, more or less. Take the treatment of diphtheria, for instance—tincture of iron, quinine, alcohol, mercury, sulphur, hyposulphites, turpentine, hydrate of chloral. Each one of these medicines has its advocates. Which one is not germicidal? Every one of them would preserve tissues, prevent putrefaction. How many diseases do we treat without using mercury, alcohol or cinchona at some time during their course? Give me three better antiseptics and I will agree that the germicidal theory is not rational.

Opium has never been proven to be germicidal, nor, indeed, am I aware that it is supposed to be so; but I predict, when we know more of the life, habits, etc., of microbes, that it will be found that this drug paralyzes or stupefies them, thus permitting and helping ever conservative nature to throw them off.

Results seem to show that Dr. Friere, of Rio Janeiro, has isolated the yellow fever germ. Of 6,524 persons inoculated by him in Rio during 1885 and 1886, eight (8) died from yellow fever. During the same period one thousand six hundred and sixty-seven (1,667) unvaccinated persons died. A large majority of the vaccinated persons were foreigners, unacclimated persons, most subject to the disease. The facts are so suggestive that comment is superfluous. Is his fate to be that of Jenner?

Diogenese, the Greek philosopher, said all men had two reasons for their actions: one was the reason which they gave; the other was the reason they did not give. Now, the reasons which are given in opposition to antiseptics are more theoretical, in my opinion, than the germ theory. The reasons which they do not give, which they keep to themselves, are, first, they do not wish to believe; and, second, they will not take the trouble to investigate.

What good has resulted to the practice of medicine from the germ theory? is often asked. As yet, practically none, so far as treatment is concerned, but the future will bring forth. Small-pox is the first in a long list of preventable diseases that science will practically banish. An ounce of preventive is better than a pound of cure. Knowing the true cause will enable us to prevent effect.

